# Genetic and Environmental Influences on Decoding Skills – Implications for Music and Reading

**DOI:** 10.3389/fpsyg.2019.02604

**Published:** 2019-11-19

**Authors:** Tracy M. Centanni, D. M. Anchan, Maggie Beard, Renee Brooks, Lee A. Thompson, Stephen A. Petrill

**Affiliations:** ^1^Department of Psychology, Texas Christian University, Fort Worth, TX, United States; ^2^The University of Alabama, Tuscaloosa, AL, United States; ^3^The Ohio State University, Columbus, OH, United States; ^4^Case Western Reserve University, Cleveland, OH, United States

**Keywords:** reading, music, genetics, education, passage comprehension, decoding

## Abstract

Music education is associated with increased speech perception abilities and anecdotal evidence suggests musical training is also beneficial for performance in a variety of academic areas. In spite of this positive association, very little empirical evidence exists to support this claim except for a few studies linking musical training to improvements in verbal tasks. We evaluated the relationships between specific aspects of musical training/ability and scores on a series of standardized reading assessments in a sample of twins. There was a significant and positive relationship between self-reported sight-reading ability for sheet music and performance on passage comprehension – a standardized reading measure that relies on decoding and working memory. This effect was specific to sight reading ability, as other musical variables, such as number of years of practice or music theory, were not related to performance on this reading measure. Surprisingly, the verbal working memory ability we tested did not mediate this relationship. To determine whether there is a genetic component to these skills, we compared these relationships in pairs of monozygotic twins compared to dizygotic twins. Interestingly, intraclass correlations (ICCs) for sight reading and passage comprehension were both higher in monozygotic twins compared to dizygotic twins, though this effect was larger for passage comprehension than for sight reading. These results together suggest a familial and potentially partially shared inherited mechanism for success in both musical sight-reading ability and passage comprehension.

**Public Significance Statement:** This study demonstrates a potentially shared, biological mechanism, subserving both basic reading skills as well as musical sight reading. Adolescents who were able to sight read at a high level, per self-report, scored higher on a measure of reading comprehension and decoding. These findings have implications for both music education and reading instruction.

## Introduction

The link between musical training and academic performance is not clearly understood, despite decades of research. As a result, music education is often one of the first departments to lose funding when school budgets are tight. Several studies have reported a positive relationship between musical training and academic achievement, though these findings are not universally reported (Douglas and Willatts, 1994; Morrison, 1994; Gardiner et al., 1996; Kelstrom, 1998; Haley, 2001; Schellenberg, 2004; Trainor et al., 2009; Cabanac et al., 2013; but see Rafferty, 2003; Costa-Giomi, 2004). Contradictory findings across these studies may be the result of differences in experimental design (Butzlaff, 2000) including in the musical instrument studied (Cheek and Smith, 1999), the intensity of training (Whitehead, 2001), or in the individuals’ self-efficacy – a person’s belief that they have the skills necessary to successfully complete an action (McPherson and McCormick, 2006).

A growing body of empirical evidence suggests that musical training and experience have positive effects not just on speech and auditory processing (Magne et al., 2006; Hyde et al., 2009; Kraus and Chandrasekaran, 2010), but on cognitive and reading tasks as well. Musical training improves verbal memory after brief (20 days of training; Moreno et al., 2011) as well as prolonged training periods (13–15 months; Schlaug et al., 2005; Forgeard et al., 2008; Jakobson et al., 2008; Roden et al., 2012). Musical training also increases the neural response to syntax errors in spoken sentences (Jentschke and Koelsch, 2009). Children who received musical training before the age of 15 years exhibit better verbal memory compared to those without musical instruction (Chan et al., 1998; Ho et al., 2003). Children who continued their musical training, as well as members of the control group who began training later, exhibited these verbal memory gains a year later (Ho et al., 2003; Rickard et al., 2010). Compared to non-musicians, only trained musicians showed gains in long-term verbal memory and verbal working memory. However, musicians lost this advantage when articulatory suppression was used to disrupt verbal rehearsal (Franklin et al., 2008). In addition to verbal memory improvements, the length of musical training predicted reading comprehension in 6- to 9-year-old typically developing readers (Corrigall and Trainor, 2011). This result persisted despite controlling for variables such as age, socioeconomic status (SES), auditory perception, IQ, number of hours spent reading per week, and word decoding skills.

Mediating factors or genetic links could also be responsible for variation in cognitive benefits of musical training. Just as certain shared skills necessary for both reading and math performance are correlated and heritable (Harlaar et al., 2012; Willcutt et al., 2013), there may also be a shared genetic component driving reading and musical ability. For example, a twin study demonstrated the heritability of pitch perception (Drayna et al., 2001), supporting the hypothesis that musical skill has at least some genetic basis. Such findings demonstrate the heritability of one specific and foundational musical skill and suggest that other musical traits may also have a genetic component.

Common brain mechanisms underlie the development of reading and music abilities (Strait et al., 2011) so it is not surprising that a number of studies have investigated the use of music therapy for a variety of language impaired groups including children at risk for reading impairment (Kraus and Chandrasekaran, 2010) and those suffering from speech production deficits after stroke (Altenmüller and Schlaug, 2013). Recent evidence suggests that there are homologous neural responses marking errors in syntax in speech and errors in chord structure in music and that musical training can enhance these speech error responses (Jentschke and Koelsch, 2009), further supporting the hypothesis that these systems interact and that training in one may influence the other. However, it is unknown whether the benefits of musical training also apply to improvement on reading tasks. Reading is a cultural invention without a dedicated brain network from birth. Over the course of experience and training, the reading network develops, most often in the left-hemisphere, and interacts heavily with the existing language network. It is currently unknown whether musical training in childhood may benefit reading ability or if there is some inherited trait that predisposes an individual to be good at both skills. To address this question, the aim of the current study was to evaluate the link between musical training and reading performance in a group of monozygotic and dizygotic twins by evaluating whether musical training was (1) associated with improved reading performance, (2) whether domain-general executive functions such as working memory mediated these skills, and (3) whether there was evidence of shared heritability for these two skills. We evaluated the relationship between various aspects of musical training, experience, and perceived skill with scores on a standardized battery of reading and working memory assessments in a sample of adolescent twins.

## Materials and Methods

### Participants

As part of a larger study on language and math development, 118 children (59 pairs of twins; 29 of which were monozygous) were recruited (56 female). Zygosity was confirmed using saliva samples collected from the majority of participants, although a questionnaire measure of twin similarity was employed when families did not wish to provide a saliva sample (Nichols and Bilbro, 1966). In the larger study, participants were followed longitudinally over 10 time points, beginning in elementary school. At each assessment, parents and children both completed questionnaires and children were tested on a battery of standardized assessments targeting language, reading, math, working memory, and other executive functions. These assessments varied by age and grade level. The assessment battery is described in additional detail below. In the current study, we considered only data collected at the final time point. At this time point, participants were 17.15 ± 1.72 years old.

### Assessment Battery and Musical Questionnaire

All children completed several subtests measuring reading, including the Woodcock Reading Mastery Test- Revised: Letter Identification, Word Attack, and Passage Comprehension subtests (Woodcock, 1998) and the Test of Word Reading Efficiency: Sight Word Efficiency and Phonemic Decoding Efficiency subtests (Torgesen et al., 1999). Participants also completed a working memory measure. Sentence Span was used to assess working memory in the verbal domain (Siegel and Ryan, 1989). Children listened to a series of sentences that were missing the terminal word and were instructed to fill in the blank. After several such sentences, children were prompted to recall all the missing words from the series, in order. At the end of each set, an additional sentence was added to the series to make the series longer and more difficult until the child failed the series or reached six sentences per series. To assess each participant’s musical training history, a six-question survey was administered to each child. Questions probed the level of musical training received in the form of lessons, time spent practicing, music theory knowledge, and sight reading. Questions used are displayed in Table 1. Since SES is strongly related to academic achievement (Bradley and Corwyn, 2002; Krapohl and Plomin, 2016), we also calculated an SES composite score based on parental education level and household income.

**TABLE 1:** Music questions administered to each child.

(1) Do you play an instrument? Circle one. YES NO a. If YES, which one(s) and how many years have you played each?
(2) Have you ever taken lessons on an instrument? Circle one. YES NO a. If YES, how many years?
(3) Have you played in any ensembles/concert bands/jazz bands/etc.? Circle one. YES NO a. If YES, which groups and for how long?
(4) Have you ever played in a personal band (e.g., rock band with friends, etc.)? Circle one. YES NO a. If yes, what instrument did/do you play, and how many years have you been part of this group?
(5) Have you ever taken music theory lessons? Circle one. YES NO If yes, for how many years?
(6) Rate your ability to read music on a scale of 1–5 (1-can’t read music, 3-can read music, 5-can sight read music). Circle a number: 1 2 3 4 5

### Statistics

To evaluate the effects of musical training on cognitive tasks, Pearson’s *r* was used with two-tails. For any measure that demonstrated consistent ceiling performance, Spearman’s rho was used to account for this skew in scores (marked as *R*_*s*_). Intraclass correlations (ICCs) were used to evaluate the effect of genetics on these relationships, comparing within the group of monozygotic or dizygotic twins. We ran two sets of analyses using this method. First, we compared scores on the same measures within twin pairs to evaluate the role of genetics and the environment on a single trait (passage comprehension or sight-reading). We then ran these analyses to cross-correlate across both measures within each twin pair (e.g., passage comprehension scores from twin A vs. sight-reading rating from twin B). Since including both twins in each pair may bias the findings, we also ran all analysis, except for the ICC, using just one twin in each pair. Bonferroni correction was used to correct for multiple comparisons.

## Results

There were no significant differences between monozygotic and dizygotic twins on any of our measures (independent *t*-tests, *p* > 0.05; see Table 2 for group means and individual *t*-tests). There were significant differences on several measures between the group of participants with any level of musical training (*N* = 87) compared to those without any musical training (*N* = 31). Those with musical training scored significantly higher than their non-musically trained peers on all reading measures (see Table 2 for group means and individual *t*-tests) except Word ID [*t*(116) = 1.43, *p* = 0.15, two-tailed].

**TABLE 2:** Participant scores on reading, music, and SES measures across groups.

	**Monozygotic twins (*N* = 58)**	**Dizygotic twins (*N* = 60)**	**MZ vs. DZ *t*-values**	**With musical training (*N* = 87)**	**Without musical training (*N* = 31)**	**Music training vs. no music training *t*-values**
Passage comprehension	105.21 ± 8.92	103.17 ± 9.08	1.23	105.79 ± 8.89	99.61 ± 7.85	**3.42**^∗∗∗^
TOWRE SWE	100.76 ± 11.72	98.93 ± 11.24	0.86	101.40 ± 11.43	95.52 ± 10.57	2.50^∗^
TOWRE PDE	96.21 ± 8.57	94.90 ± 11.02	0.71	96.82 ± 8.99	91.97 ± 11.40	2.39^∗^
WRMT WA	94.79 ± 7.37	95.47 ± 12.15	0.36	96.41 ± 10.06	91.55 ± 9.28	2.36^∗^
WRMT WID	96.16 ± 6.24	97.57 ± 7.21	1.14	97.40 ± 6.17	95.39 ± 8.12	1.43
Years of instrumental lessons	2.48 ± 3.48	2.48 ± 3.07	0.001	3.37 ± 3.38	0 ± 0	**5.53**^∗∗∗^
Years of theory training	0.39 ± 1.46	0.42 ± 1.73	0.07	0.55 ± 1.84	0 ± 0	1.66
Sight-reading ability	2.66 ± 1.48	2.88 ± 1.25	0.91	3.28 ± 1.19	1.35 ± 0.066	**8.53**^∗∗∗^
SES	7.18 ± 1.51	5.71 ± 1.47	**4.73**^∗∗∗^	6.39 ± 1.83	6.48 ± 1.04	0.22

*Socioeconomic status (SES) was calculated as the average parent education level (a Likert scale between 1 and 8) and household income (a Likert scale between 1 and 14). Higher values indicate higher SES (possible range = 1–11). All measures are reported as mean ± standard deviation. Two comparisons were made – between participants in a monozygotic pair (*N* = 58) vs. participants in a dizygotic pair (*N* = 60) as well as between participants with some degree of musical training (*N* = 87) vs. those with no musical training (*N* = 31). ^∗^*p* < 0.05, ^∗∗∗^*p* < 0.001. Bold indicates significant *p*-values that survived Bonferroni correction.*

**FIGURE 1:**
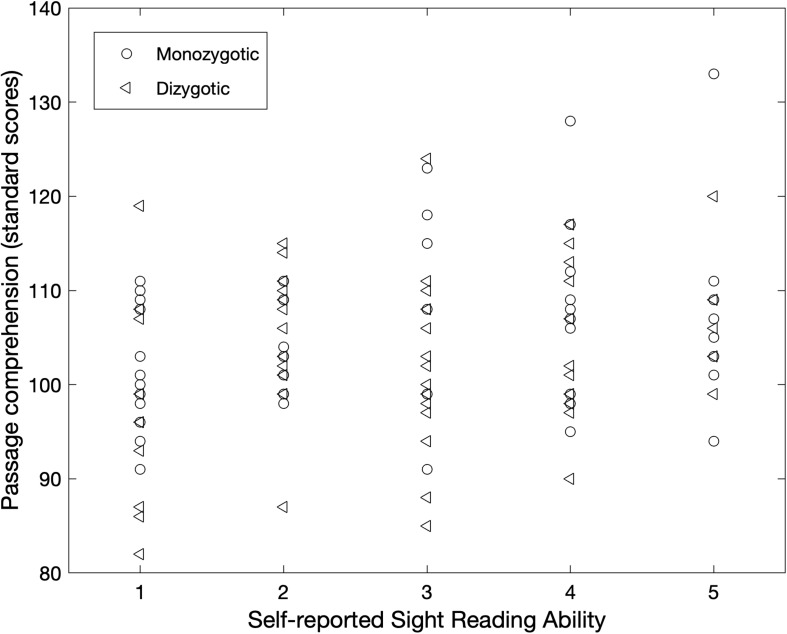
Significant correlation between passage comprehension and sight-reading. Across the entire sample, there was a significant relationship between scores on passage comprehension and self-reported sight-reading ability. Although the relationship was calculated across the entire sample, monozygotic twins are shown as circles and dizygotic twins are shown as triangles for transparency.

To determine whether musical training influences aspects of reading performance, we evaluated the relationships between several aspects of musicianship (including years of musical training, years of musical theory instruction, and sight-reading ability) with a number of reading measures. These reading measures included timed word and pseudoword reading (TOWRE-2 Sight Word Efficiency and TOWRE-2 Phonemic Decoding Efficiency), untimed word and pseudoword reading (WRMT-R Word Identification and WRMT-R Word Attack), and passage reading (WRMT-R Passage Comprehension). Years of musical training was not significantly correlated with any of the reading measures we evaluated (see Table 3 for all values). Interestingly, years of musical theory instruction also was not significantly correlated with any of the reading measures evaluated. Sight reading ability was significantly and positively correlated with passage comprehension (*r* = 0.29, *p* = 0.001; Figure 1) and with untimed word reading (*r* = 0.18, *p* = 0.04), but this latter relationship did not survive correction. There was a trend in the positive relationship between sight reading and timed pseudoword decoding (*r* = 0.17, *p* = 0.07). There were no significant relationships between sight reading and timed word reading (*p* = 0.29) or untimed pseudoword decoding (*p* = 0.12).

**TABLE 3:** Correlation values for relationships between aspects of musical training and standardized reading measures.

	**Years of instrumental lessons**		**Years of theory training**		**Sight reading ability**	
			
	***R***	***p***	***R***	***p***	***R***	***p***
Passage comprehension	0.176	0.139	0.173	0.592	**0.29**	**0.001**
TOWRE SWE	−0.042	0.728	−0.130	0.688	0.097	0.293
TOWRE PDE	−0.033	0.783	0.038	0.906	0.166	0.07
WRMT WA	−0.002	0.984	0.036	0.912	0.143	0.119
WRMT WID	0.004	0.973	0.210	0.513	0.184	0.043

*Bold indicates significant *p*-values that survived Bonferroni correction.*

To ensure our results were not due to bias introduced by including both members of each twin pair, we ran all of the above analysis again with only one twin in each pair. Sight reading ability remained significantly and positively correlated with passage comprehension (*r* = 0.28, *p* = 0.029) but was no longer correlated with untimed word reading (*r* = 0.18, *p* = 0.18). There were no significant relationships between sight reading and any other word reading measure (*p*s > 0.25).

In the passage comprehension assessment, the participant read a passage silently and filled in a missing word. This requires some degree of working memory to keep the elements of the passage in mind when determining the correct missing word. Similarly, when sight reading a piece of music, an individual must keep in working memory many elements, including the key signature, time signature, and tempo. To determine whether working memory skills underlie the relationship between sight reading and passage comprehension, we used a verbal working memory assessment as a covariate, the Sentence Span task. In this task, participants listened to a sentence with the last word missing. They were asked to fill in the blank and then hold their answer in memory. Additional sentences were added within each set and at the end of the set, the participant was prompted to recall all their answers in order. When performance on this measure was added as a covariate, the relationship remained significant (*r* = 0.290, *p* = 0.001; Table 4). Although the relationship between Word ID and sight-reading ability was nominally significant, the relationship between Passage Comprehension and sight reading remained significant when Word ID was used as a covariate (*r* = 0.228, *p* = 0.012, two-tailed). The relationship also remained significant when SES was used as a covariate (*r* = 0.221, *p* = 0.035).

**TABLE 4:** Correlation values for relationships between aspects of musical training and standardized reading measures correcting for several important variables of interest.

**Controlling for:**	**Partial correlations between sight reading and passage comprehension**	
	
	***R***	***p***
*N*-back – accuracy of trials w/response	0.304	0.001
*N*-back – reaction time	0.300	0.001
Sentence-span	0.290	0.001
WRMT WID	0.228	0.012
Socioeconomic status (SES)	0.221	0.035

**FIGURE 2:**
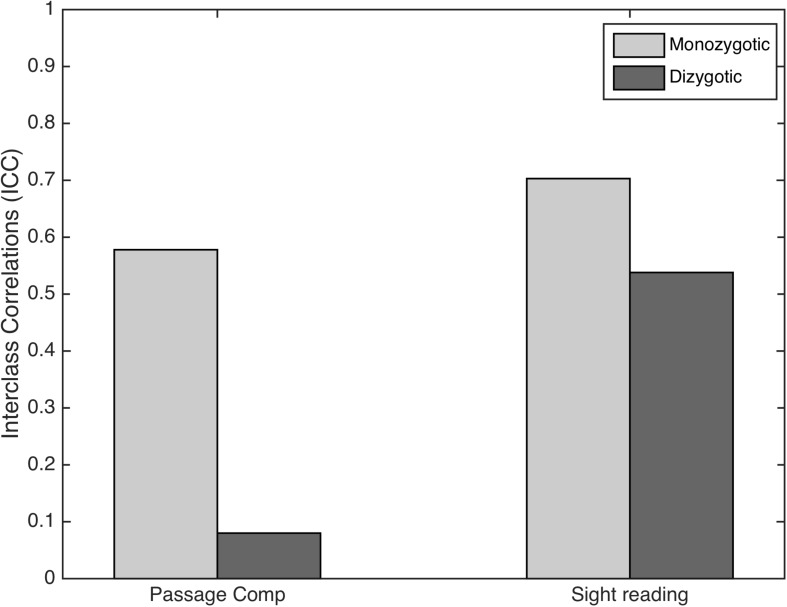
Interclass correlations (ICCs) for passage comprehension and sight-reading among monozygotic vs. dizygotic twins. The difference in ICC values was much larger in the passage comprehension compared to the sight-reading condition suggesting a strong genetic influence for passage comprehension performance and a stronger environmental influence for sight-reading.

**FIGURE 3:**
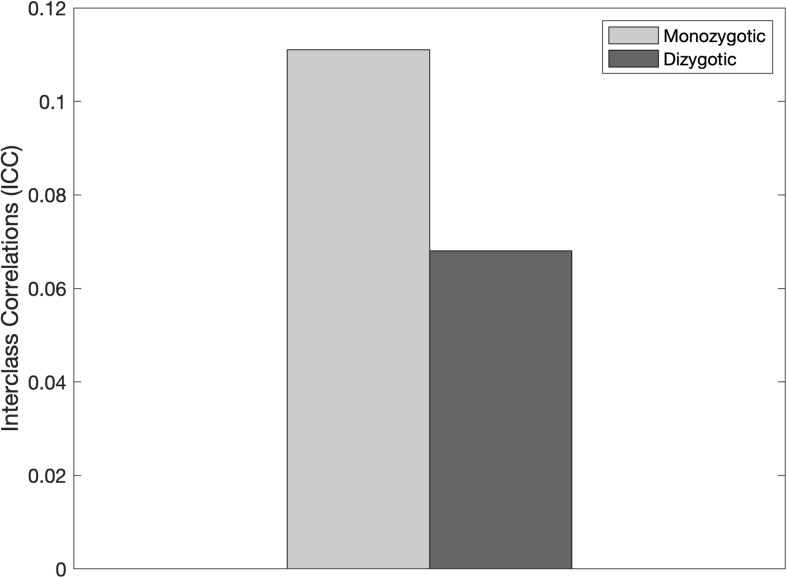
Interclass correlations (ICCs) between passage comprehension and sight-reading among monozygotic vs. dizygotic twins. The ICC values were larger in the monozygotic twins compared to the dizygotic twins, suggesting a muted genetic effect on the relationship between these two measures.

Finally, to examine whether genetic influences on musical sight reading and passage comprehension were indicated, we calculated ICCs in the subset of monozygotic twin pairs and compared them to the ICCs in the dizygotic twin pairs for each measure. For Passage Comprehension, the correlation was higher in monozygotic twins (0.578) compared to dizygotic twins (0.080; see Figure 2). For sight reading, the correlation in monozygotic twins was again higher than in dizygotic twins (MZ = 0.703, DZ = 0.538; see Figure 1). To evaluate the potential genetic and environmental influence on the relationship between these two measures we also ran interclass correlations comparing passage comprehension performance in twin A to sight-reading ability in twin B (Figure 3). This analysis revealed an ICC for monozygotic twins of 0.111 and 0.068 for dizygotic twins, suggesting low heritability for this relationship.

## Discussion

In this study, we evaluated the relationship between various aspects of musical training and reading skills in a sample of adolescent twins. The study aimed to address the genetic vs. environmental links between musical training and reading performance in a group of monozygotic and dizygotic twins. We evaluated whether musical training was associated with improved reading performance, whether this relationship was mediated by verbal working memory, and whether there was evidence of shared heritability for reading and music. We found a significant relationship between sight reading ability and passage comprehension performance. There were no relationships between passage comprehension and other music measures, suggesting a shared skill between these two measures. Verbal working memory abilities did not influence this relationship. Finally, in a comparison between monozygotic and dizygotic twins, passage comprehension demonstrated a stronger genetic component than sight reading. These findings add to the growing empirical literature on the relationship between musical training and skills relevant to academic performance and suggest a complex relationship between genetics and environment on the interaction between these skills.

### Potential Shared Skills Between Passage Comprehension and Sight-Reading

A growing body of evidence supports the link between musical training and elements of reading performance. Notably, one study reported a significant relationship between the years of musical training received and performance on passage comprehension (Corrigall and Trainor, 2011). In partial support of this finding, we observed a trend in the number of years of instrumental lessons and passage comprehension. In addition, we observed a significant, positive relationship between scores on passage comprehension and sight-reading, which the previous study did not assess. While we cannot rule out other factors that may have influenced this discrepancy in finding, such as differences in SES, rhythm processing, and home literacy environment (Corrigall and Trainor, 2011), our findings suggest that years of musical training may be less important in reading transfer as the skills learned during that training.

If sight reading is the skill that specifically mediates the relationship between musical training and passage comprehension performance, then there may be a shared skill subserving both passage comprehension and musical sight-reading. We hypothesized that working memory skills underlie the relationship between sight-reading and passage comprehension. Retaining elements of a passage in their working memory when determining the correct missing word for the passage comprehension task is similar to sight-reading a piece of music where participants must retain elements like the key signature, time signature, and tempo in their working memory. Children with high reading comprehension performance performed better on working memory for recall of digit lists (Oakhill et al., 2005), suggesting that working memory is a required skill for reading comprehension. However, other data seem to dispute this claim. After controlling for age, IQ, and single word reading, working memory contributed only 3% of additional variance toward explaining reading comprehension abilities (Goff et al., 2005).

We did not observe any relationship between our working memory measure and either passage comprehension or sight reading in our sample. One potential explanation for this discrepancy is the type of working memory measure used. Unfortunately, we did not have any measures of non-verbal working memory and so we cannot speak to whether this type of working memory may better explain the relationship between sight-reading and passage comprehension. Another possible explanation is the type of reading comprehension measure used. It is possible that measures of passage comprehension in which missing words are provided by the participant do not adequately measure reading comprehension. In support of this hypothesis, in a large sample of children and adolescent’s performance on passage comprehension was best explained by performance on decoding and further, passage comprehension ability varied by age (Keenan et al., 2008), suggesting that this measure may more accurately model decoding ability rather than true comprehension. If passage comprehension, as evaluated in our study, is influenced by decoding rather than working memory, it then makes sense that our working memory measure did not affect the passage comp–sight-reading relationship.

Decoding is a required skill for both passage reading and music sight-reading. When an individual sight reads, they must quickly and efficiently recognize notes in the score and link them with the correct actions needed to produce the note. Further, if the key signature requires a different action in a certain context, the musician must be able to quickly recognize this and adapt (for a review on sight reading and its components, see Gudmundsdottir, 2010). For example, if the key signature indicated B ♭ (B flat), then every time the musician sees the note B, they must adjust their response accordingly to produce a slightly different note. This skill is also critical in reading, especially in English, where individual letter combinations may have different pronunciations depending on their context. Of the decoding measures we used (TOWRE Sight Word Efficiency and Phonemic Decoding Efficiency as well as WRMT Word ID and Word Attack), only Word ID was correlated with sight reading and even this relationship did not survive correction. It could also be the case that poor decoding at a more basic level is responsible for the shared variance between these two measures. The brain develops reading fluency over a long trajectory, lasting into adulthood (Centanni et al., 2017). Once fluency is achieved, print symbols are quickly and effortlessly decoded and integrated into the text as a whole and reading that symbol. The same is required in sight reading – quickly and effortlessly decoding the printed note into the melody as a whole and executing that note. Evaluation of this hypothesis requires a measure of fluency, often measured using a rapid automatized naming task, which we did not collect from participants at this time point. However, future research should investigate this possibility.

One other possibility is visual perceptual span – the amount of information that can be extracted and used around a given fixation point. In a simple paradigm where no complex key signatures were presented, musicians who were most accurate at sight-reading a piece of piano music had a longer eye–hand span, meaning that while playing, they were better able to take in visual information farther in the future from the current note (Truitt et al., 1997). In sight reading music, a visual symbol must be quickly translated into the appropriate motor movements necessary to produce the correct note. Since music is played at a specific tempo, the eyes usually lead the hands – the musician is looking a few notes ahead of what they are currently playing (Kinsler and Carpenter, 1995; Rayner and Pollatsek, 1997). In reading out loud, a similar pattern emerges: the eyes bring in a visual stimulus that elicits a motor response and the eyes tend to fixate just ahead of what the voice is currently doing (for review, see Rayner, 1998). It is therefore possible that visual span is the skill subserving both passage comprehension and sight reading. In the current study, we did not have a measure of eye span, so future studies are needed to better answer this question.

### Heritability of Music Sight-Reading and Passage Comprehension

In twin studies, performance on several cognitive tasks are correlated with each other, suggesting a shared genetic trait necessary for both domains. Math and reading, for example, are strongly related such that high performance on math assessments is correlated with high performance on reading assessments (Kovas et al., 2005). This relationship may be mediated by attention, which appears to be a shared skill necessary for both reading and math (Peterson et al., 2017). The observation that certain sets of cognitive skills are correlated in sets of twins suggest a genetic basis. The Generalist Genes Hypothesis states that there may be certain genes that control these shared skills known as generalist genes (Plomin and Kovas, 2005). This hypothesis was directly tested by evaluating a series of small genetic mutations known as single-nucleotide polymorphisms (SNPs) that are associated with reading disability (Haworth et al., 2007). Specific genotype profiles across all SNPs tested were significantly correlated with not just reading skills, but math abilities (such as numbers and algebra) and general cognitive skills (such as vocabulary, similarity, and concept grouping; Haworth et al., 2007).

The difference in ICC in our study between monozygotic twins and dizygotic twins was several times larger for passage comprehension than for sight-reading. Our sample size was too small to make meaningful comparisons of effect size across conditions. If this effect is replicated in a larger sample, it would suggest that developing skill in music sight-reading may rely heavily on environmental factors, such as instructor quality and learning environment. If this is confirmed, the link between sight reading and passage comprehension could therefore be directional in nature: it may be the case that inherited abilities related to passage comprehension may provide an advantage for sight-reading beyond that provided by genetics, such as eye span. This hypothesis is supported by the dampened difference in ICC between MZ and DZ twins when both traits were considered. This dampening, however, may also be due to better controlled psychometric properties in the passage comprehension measure and a lack of empirical testing of sight-reading ability. Unreliability in a measure’s validity would dampen the differences between MZ and DZ correlations.

Unfortunately, due to the small sample size in our study, we were unable to run structural equation modeling to evaluate the nature of this relationship (Rijsdijk and Sham, 2002). Further, the small difference in ICCs for sight reading and the even smaller ICCs for the relationship between passage comprehension and sight reading suggest that structural equation models are unnecessary. However, due to the limited power in the current study, future research is needed to evaluate this hypothesis and test the role of environment vs. genetics on the relationship between sight reading and passage comprehension using more advanced methods, like SEM.

### Study Limitations and Future Directions

There are a couple of limitations in the current study to note. First, our sample was under-powered for a twin study so it is likely that our results were influenced by some amount of random fluctuation. Further, due to our small sample size, we included both twins in each pair, which may have influenced the strength of the relationships we report here. When we ran the analyses with a smaller sample including just one twin in each pair, the main finding remained significant. However, these findings should be interpreted as preliminary and future research should investigate the relationship between sight reading and passage comprehension with a larger, independent sample. Our sample size also precluded us from conducting structural equation modeling analyses, as discussed above. Second, our knowledge of each child’s sight-reading ability was acquired through self-report, which is often not accurate. A child may over- or under-estimate their ability, or not understand what “sight-reading” entails, which could bias our findings. To confirm the relationship observed here, future studies are needed in which a more objective measure of sight reading is obtained, either through their current music teacher or by testing them directly in the lab to get a more accurate measure. Third, we did not run a randomized training trial in which participants were randomly assigned to music training or some type of control training. For this reason, the direction of the relationship between passage comprehension and sight reading cannot be determined using our results. Future research is needed where participants are randomized into music training or delayed-start control training to better evaluate whether sight reading ability improves passage comprehension skills.

If it is determined that musical training, and specifically sight-reading ability, is beneficial for passage comprehension, this would support future research on the use of musical training as a therapy tool for those struggling with early reading difficulties. A study on the effect of music vs. painting training on language and reading demonstrated improvements on speech perception tasks as well as a rapid word reading task in the group who received music training (Moreno et al., 2009). Music training has also been associated with improvement on phoneme awareness and naming speed performance in second language learners (Herrera et al., 2010) and improved neural responses to semantic errors in language (Jentschke and Koelsch, 2009). However, additional studies are needed to determine the direction of this relationship using a randomized approach to control for musical training. Our findings demonstrate, for the first time, a link between music sight reading ability and passage comprehension skills, and suggest that this skill, while not highly heritable, may provide additional benefit to the learner in other domains.

## Data Availability Statement

The datasets generated for this study are available on request to the corresponding author.

## Ethics Statement

The studies involving human participants were reviewed and approved by The Ohio State University Institutional Review Board. Written informed consent to participate in this study was provided by the participants’ legal guardian/next of kin.

## Author Contributions

SP, LT, and TC designed the study. MB, RB, LT, and SP collected the data. TC and DA analyzed the data and wrote the manuscript.

## Conflict of Interest

The authors declare that the research was conducted in the absence of any commercial or financial relationships that could be construed as a potential conflict of interest.
